# Comparison of the rebuilding effects of different computed tomography scanners and reconstructive settings for the five-line sign in normal interlobular fissures

**DOI:** 10.1186/s41747-017-0022-5

**Published:** 2017-10-19

**Authors:** Anle Yu, Qun Li, Yuefu Zhan, Jinlong He

**Affiliations:** 1 Department of Radiology, First Affiliated Hospital of Hainan Medical College, 31 Longhua Road, Haikou, Hainan 570102 China; 2 Department of Pathology, Hainan Medical College, 33 Longhua Road, Haikou, Hainan 570102 China; 3 Department of Radiology, Haikou People’s Hospital, Haikou, Hainan 570208 China; 4 Department of Ecsomatics, First Affiliated Hospital of Hainan Medical College, 31 Longhua Road, Haikou, Hainan 570102 China

**Keywords:** Computed tomography (CT), Multi-slice, Five-line signs, Interlobular fissures, Maximum Intensity Projection (MIP), Image processing, Computer-assisted, Image reconstruction

## Abstract

**Background:**

Based on the images generated from two multi-slice computed tomography (CT) scanners, we intended to compare the five-line sign of normal interlobular fissures produced on axial or oblique maximum intensity projection (MIP) reconstructions using different algorithms.

**Methods:**

Two groups of 50 subjects underwent either 16-slice or 256-slice spiral unenhanced chest CT. None of them in either group displayed any abnormality. For each case, maximum intensity projection (MIP) data were used to calculate the axial or oblique projection using four algorithms: standard axial, standard oblique, high-resolution axial, and high-resolution oblique algorithm. The results were then used to reconstruct images of six locations of the lung. The clarity of the five-line sign of the reconstructed MIPs for the interlobular fissures was determined and graded as 1 (unclear), 2 (barely clear), or 3 (clear). Comparisons of the rate and the degree of clarity were performed using non-parametric tests.

**Results:**

Data from both the 16-slice and 256-slice CT revealed that the standard oblique algorithm was the best among the four methods for presenting clear images of the five-line sign (*p* < 0.001), whereas the high-resolution axial algorithm was the worst. In addition, the two CT units exhibited no significant differences in the clarity of the five-line sign (*p* = 0.273).

**Conclusions:**

The standard oblique algorithm was the best approach to reveal the five-line sign of normal lung fissures. Both 16-slice and 256-slice CT were effective for reconstructing the sign.

## Key points

- Both 16-slice and 256-slice CT were effective for reconstructing the five-line sign of normal interlobular fissures
- The standard oblique algorithm was the best approach to reveal the five-line sign
- The MIP-rendered five-line sign has smooth lines and clear gaps and can, therefore, be used as the referencing object for other planar structures in the lung

## Background

Multi-slice spiral computed tomography (CT)-based thin reconstructed images facilitate the clear presentation of various structures and lesions in the lung from any angle [1–3]. Reconstructions based on the maximum intensity projection (MIP) algorithm can reveal the highest level of continuity of linear, high-density vascular structures in the lung. Therefore, this is an excellent method to differentiate vascular sections and small pulmonary nodules on a thin-section image [4, 5].

Using a 64-slice spiral CT scanner, we previously noticed that normal interlobular fissures presented as multiple parallel shadow lines in MIP-reconstructed images, while the number of shadow lines was equal to the ratio of the thickness of the MIP reconstruction layer to the thickness of the original layer. A five-line sign could be generated if the following parameters were adopted: 1.25-mm layer thickness, 1.25-mm space, and 6.25-mm MIP slab thickness [6]. The five-line sign of interlobular fissures could be used to differentiate the underdeveloped interlobular fissures and the accessory fissures from pulmonary linear fibrosis.

In this study, we examined the effect of two different CT units (16-slice or 256-slice), using 1.0-mm reconstruction layer thicknesses, 1.0-mm space, and also added a high-resolution reconstruction algorithm on the clarity rate and level of the five-line sign.

## Methods

### Patients and imaging methods

This retrospective study was approved by the institutional review board (Haikou People's Hospital and First Affiliated Hospital of Hainan Medical College) with a waiver of informed consent. Candidates were selected based on a consecutive series of apparently normal unenhanced CT examinations performed from December 2012 to April 2013. We included 128 subjects who had no abnormalities on a spiral chest CT scan. Among these, 62 were scanned using 128-row 256-slice CT; 12 of these subjects who had poor-quality images (n = 9) or underdeveloped interlobular fissures (n = 3) were excluded from the study. The remaining 50 subjects included 35 men and 15 women aged from 18 to 77 years. Sixty-six individuals were scanned using a 16-row 16-slice CT unit; 16 of these subjects who had poor-quality images (n = 12) or underdeveloped interlobular fissures (n = 4) were excluded from the study. The remaining 50 subjects included 28 men and 22 women aged from 18 to 75 years. Here, underdeveloped interlobular fissures means that any segment of the oblique fissures or the major, such as upper, middle or lower one was absent; poor image quality means that there was movement artefact of the bronchi, vessels, fissures and diaphragm on coronal reconstruction in the lungs.

The 256-slice CT unit was a Philips (Philips Healthcare, Amsterdam, Netherlands) Brilliance iCT (Cleveland, OH, USA). Technical parameters included a collimation width of 128 × 0.625 mm, a rotation speed of 0.75 s, and a pitch of 0.993. Image reconstruction was performed using two kernels: the standard kernel for the standard algorithm and the lung-enhanced kernel for the high-resolution algorithm. The 16-slice unit was a Siemens (Siemens Healthcare, Erlangen, Germany) Somatom Sensation 16 (Munich, Germany). Technical parameters included a collimation width of 16 × 0.75 mm, a rotation speed of 0.5 s, and a pitch of 1.1. The scanner was equipped with two kernels: the B40f kernel for the standard algorithm and the B80f ultra-sharp kernel for the high-resolution algorithm. Both CT units were operated under the following common parameters: voltage 120 kVp; tube current 200 mAs (with tube current modulation active on the 16-slice CT unit); 1.0-mm reconstruction slice thickness; 1.0-mm space; 5.0-mm axial MIP thickness; 6.0-mm oblique MIP thickness; and lung visualisation window (width 1000 Hounsfield units (HU), level − 700 HU).

### Image analysis

For multi-planar MIP reconstruction, the 1-mm thin-section-based reconstruction data from the 100 patients were imported into a Brilliance™ Workspace Portal (V2.6.1.5, Philips Medical Systems, Veenpluis 4-6, 5684 PC Best, Netherlands) to perform multiplanar and MIP reconstruction.

**Fig. 1:**
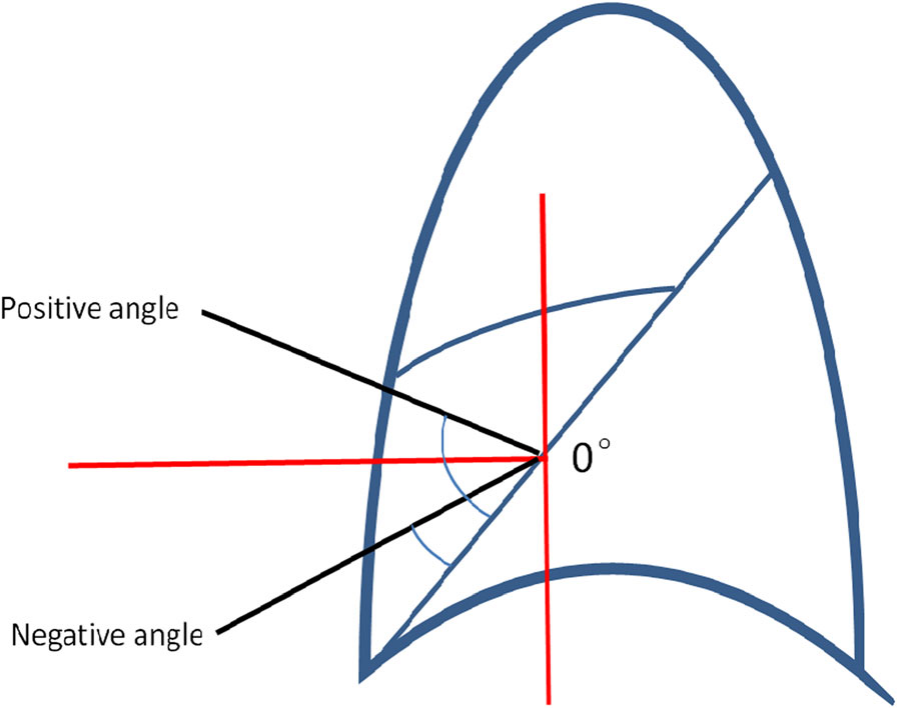
Sagittal diagram of oblique maximum intensity projection rebuilding to observe the five-line sign. The angle between the fissure and *tangent line* is positive when the *tangent line* is rotated clockwise from 0°; conversely, the angle is negative. The *long oblique line* represents the major fissure, and the short one represents the minor fissure

**Fig. 2:**
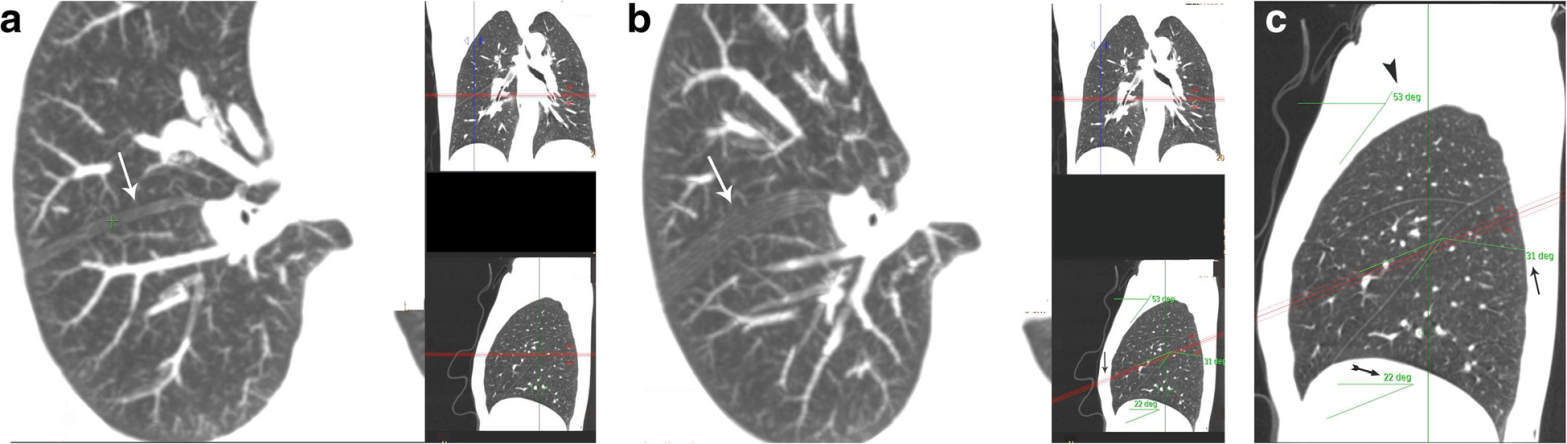
Images from 256-slice spiral computed tomography (CT). **a**-**c** Standard algorithm-generated images. Width of the lung window, 1000 Hounsfield units (HU); window level, − 700 HU. **a** At the workstation three-dimensional interface, the *left* image is an axial maximum intensity projection (MIP) reconstruction with 5-mm slab thickness showing the *lower right* oblique fissure. The interlobular fissure manifested a band-like pattern and had high density and a clear boundary. No five-line sign was revealed (*white arrow*). The basic point (*small green cross*) was set at the middle point of the *lower right* oblique fissure, coronal (*right upper* image), and sagittal plane (*right lower* image) corresponding to the middle point of the axial lower oblique fissures. **b** In the sagittal position (*right lower* image), which is the same one as shown in **a**, and the workspace interface, the reconstructed *tangent lines* were rotated counter clockwise to a given angle (*black arrow*) to allow formation of an oblique coronal plane (*left* image). For the interlobular fissure of this oblique plane, a five-line sign was clearly revealed (*white arrow*) with a MIP slab thickness of 6 mm. **c** On the sagittal position, which is the same one as the *right lower* image shown in **b**, the angle between the *tangent lines* and the oblique fissure (*arrow*) is 31°; the angle between the *horizontal line* and the oblique fissure (*arrowhead*) is 53°; and the angle between the tangent lines and the *horizontal line* (*arrow* with a fish tail) is 22°. Notice that the minor fissure is imaged as an arc line-like pattern on the sagittal plane

Two orientations were adopted for MIP reconstruction: axial and oblique (oblique coronal). Specifically, reconstruction tangent lines were rotated clockwise or counter clockwise on the sagittal plane until the five-line sign of the pleural segment was clearly displayed on the MIP-reconstructed coronal oblique plane. The angle between the fissure and the tangent line was positive as the tangent line was rotated clockwise from 0°; otherwise, the included angle was negative as the tangent line rotated counter clockwise from 0°; “+” and “-” was marked apart and each of them represented a given direction of the tangent line rotated initially, but did not indicate how small or large the degree of the angle was (Figs. 1 and 2). Bilateral interlobular fissures were divided into upper, middle, and lower zones to be observed. In the right lung the upper segment was above the intersection of the minor with the major fissure, the minor itself as the middle one, and the lower one below the intersection. Since the minor was absent in the left lung, the major fissure of the left lung was divided into the upper, middle, and lower segments for imaging observation. This was also applied to the right lung when the minor fissure was undeveloped or absent.

Each sample was subject to calculation using the standard axial algorithm, the standard oblique algorithm, the high-resolution axial algorithm, and the high-resolution oblique algorithm to reconstruct images at six locations in the lung: upper left, upper right, middle left, middle right, lower left, and lower right. Two physicians, Y.Z. and A.Y., with 5 years and 20 years of diagnostic imaging experience, respectively, worked in consensus to formulate an image clarity standard for the five-line sign of interlobular fissures in the MIP-reconstructed images.

**Fig. 3:**
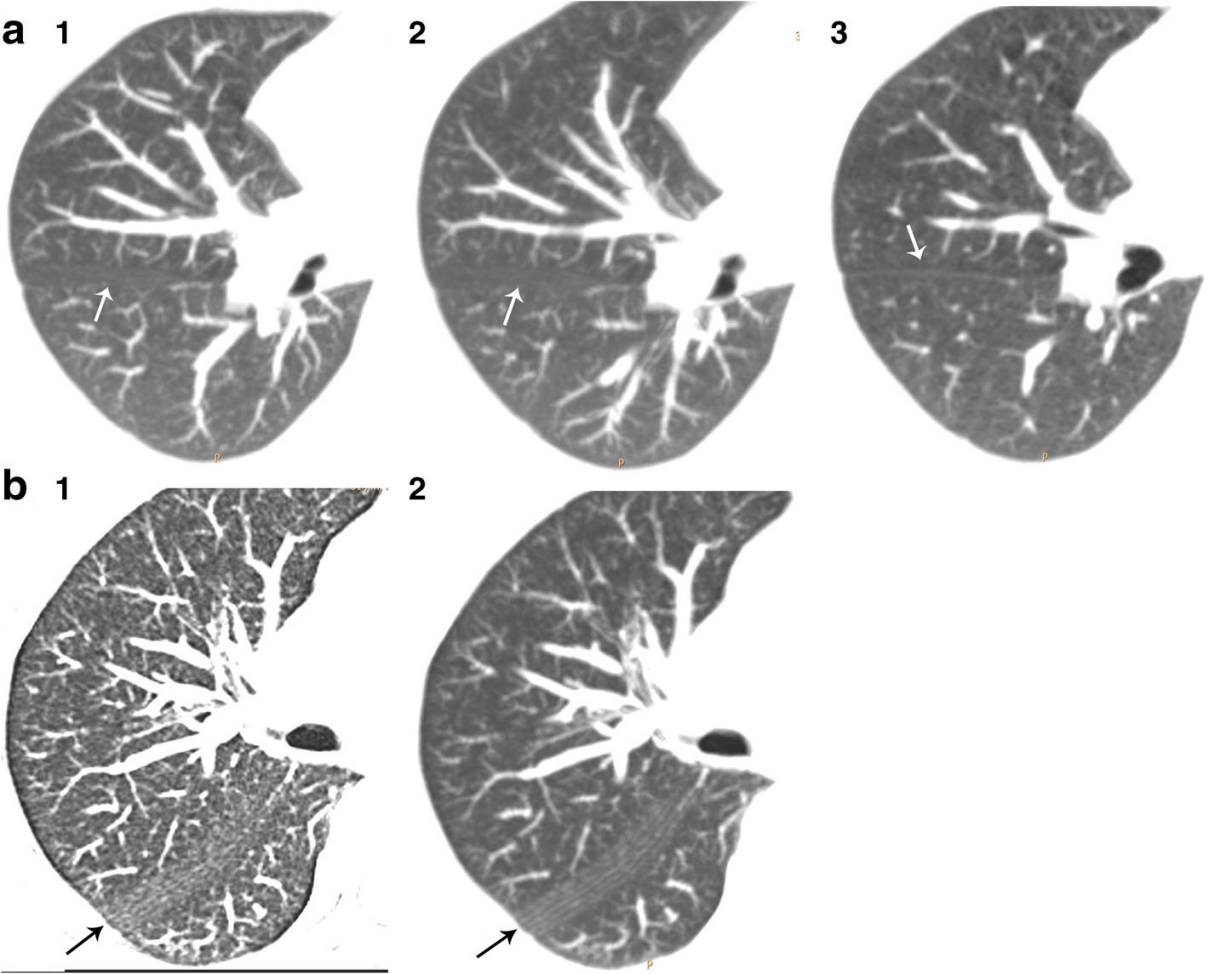
**a** Images from 256-slice spiral computed tomography (CT). **a1-3** Standard-algorithm-generated images obtained from the same individual. Width of the lung window, 1000 Hounsfield units (HU); window level, − 700 HU. **a1** Axial maximum intensity projection (MIP)-rendering with a 5-mm slab thickness showing the lower segment of the right major fissures as a low-density band shadow with a blurred edge. No five-line sign was visible (*white arrow*). **a2** MIP reconstruction at the same location as **a1** except the scan was from an oblique coronal position. The five-line sign of the lower segment of the right major fissures was still unclear (*white arrow*). **a3** Axial 1.0-mm thin-section image of the same location as in **a1** and **a2**. The lower segment of the right major fissure is manifested as a low-density *line* with a blurred edge (*white arrow*). **b** Images from 16-slice spiral CT. **b1** and **b2** Transverse images from the same individual; width of the lung window, 1000 HU; window level: − 700 HU. **b1** Axial MIP reconstruction with 5-mm slab thickness and a high-resolution algorithm. The five-line sign of the upper segment of the right major fissure was barely clear, and some *lines* were visible (*black arrow*). **b2** Same location as in **b1**; axial MIP reconstruction with 5-mm slab thickness and the standard axial algorithm. The five-line sign of the upper segment of the right major fissure was clearly presented (*black arrow*)

Based on this standard, the axial and oblique rendering outcomes of the 100 subjects who had normal interlobular fissures were assessed. The clarity levels of the five-line sign were graded as 1 (unclear, when single lines of interlobular fissures were visible in the original thin-section images, but the MIP-reconstructed image failed to reveal multiple lines and instead displayed band shadows with either a clear or an unclear boundary), 2 (barely clear, when the five lines were distinguishable but with low density, inadequate contrast and blurring gaps), or 3 (clear, when the five lines presented with distinct boundaries) (Figs. 2 and 3). For each controversial case, a third-party expert (Z.H.) with 5 years of experience in diagnostic imaging was consulted until a consensus was reached.

### Statistical analysis

Data analysis was performed using SPSS 16.0 software (Chicago, USA). The Kruskal-Wallis test was used to compare the clarity levels of the five-line sign and the angles between the fissures and tangent lines derived from the two CT units and the four calculation methods. The Kruskal-Wallis test and a subsequent Mann-Whitney *U* test were used for multiple comparison analysis of the clarity levels. The χ-square test was used to compare the rate of assignment to clarity level 3 and combined (2 + 3) clarity levels for the four calculation methods and the two CT units as well as the clarity rates of the five-line sign among six locations on axial or oblique positions. The significance level of the statistical analyses was set as α = 0.050. Inter-observer agreement for clarity levels of the five-line sign from different CT scanners and different algorithms was estimated by using weighted κ statistics [7].

## Results

**Fig. 4:**
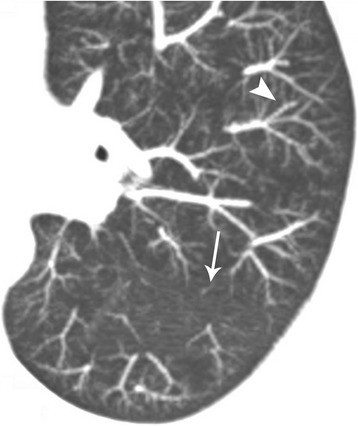
Images from 16-slice spiral computed tomography (CT). The standard algorithm generated transverse images: width of the lung window, 1000 Hounsfield units (HU); window level, − 700 HU. The 12-mm-thick axial maximum intensity projection-rendered image of the upper segment of the *left* major fissure shows decreased density of multiple *shadow lines* and the surrounding structures overlapping with multiple *shadow lines*, with most of the shadow lines being no longer visible (*white arrow*). Segmented small blood vessels are visible (*white arrowhead*)

The following settings were used for both CT units: 1.0-mm reconstruction thickness, 1.0-mm space and 5.0-mm MIP slab thickness. Correspondingly, both units generated MIP reconstruction images at the axial position that revealed parallel five-line shadow lines for the normal pleura. In other words, the number of shadow lines was equal to the ratio of the thickness of the MIP-layer to the thickness of the original layer (Fig. 3). However, when the images derived from the two units were reconstructed by oblique MIP, the workspace added 1 mm by itself to reach a thickness of 6 mm before the five-line shadow lines could be displayed. As the MIP thickness was increased, the line number doubled, resulting in a gradual reduction in the density. Consequently, the surrounding structures blended into the shadow lines, and the external lines gradually disappeared (Fig. 4).

Using 16-slice and 256-slice CT, the standard oblique algorithm presented the highest degree of clarity for the five-line sign (*p* < 0.001), whereas the high-resolution axial algorithm presented the lowest clarity (*p* < 0.001) among the four methods. There were no significant differences in the clarity of the five-line sign using the two CT devices when the same calculation method was used (Table 2). A comparison of the clarity of the five-line sign obtained from the two CT scanners using the four calculation methods is presented in Table 1.

**Table 1:** Comparison of the clarity levels of the five-line sign derived from different algorithms using 16-slice and 256-slice CT

Method	Number of sites	M (P_25_, P_75_)	
		16-slice	256-slice
HRA	300	1.00 (1.00, 1.00)^a,g,i^	1.00 (1.00, 1.00)^d,h,j^
HROA	300	2.00 (1.00, 2.00)^b^	2.00 (1.00, 2.00)^e^
SAA	300	2.00 (1.00, 2.00)^c^	2.00 (1.00, 3.00)^f^
SOA	300	3.00 (2.00, 3.00)	3.00 (2.00, 3.00)
Sum	1200	2.00 (1.00, 3.00)	2.00 (1.00, 3.00)
H		548.621	531.787
*p*		< 0.001	< 0.001

*CT* computed tomography, *HRA* high resolution axial algorithm, *HROA* high resolution oblique algorithm, *SAA* standard axial algorithm, *SOA* standard oblique algorithm. Comparison of HRA, HRO, and SAA with SOA: the *U* values of ^a^2541.50, ^b^14478.00, ^c^18292.50, ^d^2870.00, ^e^16537.00, and ^f^19508.50, respectively. Comparison of HRA and SAA: the *U* values of ^g^25832.00 and ^h^25141.50, respectively. Comparison of HRA and HRO: the *U* values of ^i^20910.00 and ^j^21283.00, respectively. The *p* value was < 0.001 for all statistical analyses

M (P_25_, P_75_) represents median (25th percentile, 75th percentile)

H in the stub represents the statistic value obtained from formula Kruskal-Wallis test. It is equivalent to the chi square value obtained by chi square test

When the standard oblique algorithm was used, the rates of assignment to clarity level 3 from six locations were 70.3% for the 16-slice CT and 74.3% for the 256-slice CT, which were not significantly different (χ^2^ = 1.199, *p* = 0.273). In the lower left zone, however, 256-slice CT yielded significantly superior results to 16-slice CT (60% versus 34%; χ^2^ = 6.784, *p* = 0.009). If data from level 2 (barely clear) were included, the total clarity rates of the six locations increased to 100% and 97.3% for 16-slice CT and 256-slice CT, respectively.

When the high-resolution oblique algorithm was used, 256-slice CT exhibited a higher rate of assignment to clarity level 3 than did 16-slice CT (48% versus 14%, χ^2^ = 13.551, *p* < 0.001) in the lower right lung area. In addition, there were no significant differences between the two CT scanners in the clarity of the five-line sign at any location when calculated using the same methods. The clarity rates of the five-line sign derived from the different methods and the two CT devices are summarised in Table 2.

**Table 2:** Comparison of the clarity level of the five-line sign derived from 16-slice and 256-slice CT

Method	Unit	Clarity level			Sum	X^2^	*p*
		1 (%)	2 (%)	3 (%)			
HRA	16-slice	253 (84.3)	44 (14.7)	3 (1.0)	300	3.514	0.173
	256-slice	268 (89.3)	29 (9.7)	3 (1.0)	300		
HRO	16-slice	96 (32.0)	168 (56.0)	36 (12.0)	300	10.736^a^	0.005
	256-slice	114 (38.0)	130 (43.3)	56 (18.7)	300		
SAA	16-slice	135 (45.0)	92 (30.7)	73 (24.3)	300	3.189	0.203
	256-slice	143 (47.7)	73 (24.3)	84 (28.0)	300		
SOA	16-slice	0 (0.0)	89 (29.7)	211 (70.3)	300	10.863^b^	0.004
	256-slice	8 (2.7)	69 (23.0)	223 (74.3)	300		

*CT* computed tomography, *HRA* high resolution axial algorithm, *HROA* high resolution oblique algorithm, *SAA* standard axial algorithm, *SOA* standard oblique algorithm

^a^Rate of clarity level 3 (16-slice versus 256-slice CT): χ^2^ = 5.135, *p* = 0.023

^b^Rate of clarity level 3 plus level 2 (16-slice versus 256-slice CT): χ^2^ = 6.208, *p* = 0.013

Table 3 shows that the five-line signs displayed directly on the axial MIP images in most of the upper segments of the right major fissures and of the minor fissures; however, the oblique lines had to be adjusted to show the five-line sign in the remaining segments of the bilateral fissures.

**Table 3:** Cases of clear or barely clear five-line sign on maximum intensity projection reconstruction in axial or oblique positions using the standard algorithm

Locations	Cases	16-slice		256-slice	
		Axial (%)	Oblique (%)	Axial (%)	Oblique (%)
Upper right	50	35(70)	15(30)	35(70)	13(26)
Middle right	50	33(66)	17(34)	40(80)	9(18)
Lower right	50	0(0)	50(100)	4(8)	45(90)
Upper left	50	5(10)	45(90)	1(2)	47(94)
Middle left	50	1(2)	49(98)	1(2)	48(96)
Lower left	50	0(0)	50(100)	0(0)	49(98)
χ^2^		153.60	153.60	177.50	169.50
*p*		< 0.001	< 0.001	< 0.001	< 0.001

Table 4 shows that there were no significant differences between the two CT scanners in the angles of the same location between the fissures and the tangent lines, but there were significant differences in the angles among different locations using the same CT scanner.

**Table 4:** Included angle between fissures and tangent lines showing a clear or barely clear five-line sign using the standard algorithm

Locations	16-slice			256-slice		
	Cases	Included angle, degrees	Range, degrees	Cases	Included angle, degrees	Range, degrees
		M (P25, P75)	(P2.5, P97.5)		M (P25, P75)	(P2.5, P97.5)
Right upper	50	− 27.00^†^ (− 31.15, − 20.75)	− 54.33 ~ − 2.10	48	− 27.50 (− 31.00, − 19.25)	− 40.87 ~ − 2.35
Right middle	50	− 0.50 (− 18.00, 17.00)	− 43.35 ~ 37.80	49	− 3.00 (− 13.00, 11.00)	− 37.50 ~ 31.00
Right lower	50	− 34.50 (− 39.00, − 31.50)	− 45.72 ~ − 23.00	49	− 33.00 (− 36.00, − 28.00)	− 44.00 ~ − 21.00
Left upper	50	− 31.50 (− 37.00, − 28.00)	− 45.72 ~ − 16.28	48	− 33.00 (− 38.50, − 29.00)	− 45.55 ~ − 20.22
Left middle	50	− 32.00 (− 36.00, − 30.00)	− 40.18 ~ − 6.42	49	− 32.00 (− 35.00, − 28.00)	− 41.00 ~ − 17.25
Left lower	50	− 36.00 (− 42.00, − 31.75)	− 46.45 ~ − 22.00	49	− 34.00 (− 37.50, − 31.00)	− 46.00 ~ − 21.25
H		113.97			125.57	
p*		< 0.001			< 0.001	

^†^“−” Indicates direction only; the absolute values of included angles represent the size

*Differences in angles between fissures and the tangent lines among six locations using the same computed tomography scanner were significant

Inter-observer agreement for the clarity of the five-line sign for the high resolution axial algorithm, the high-resolution oblique algorithm, the standard axial algorithm, and the standard oblique algorithm was represented by κ values of 0.142, 0.559, 0.679, and 0.922 for the 16-slice CT scanner and 0.125, 0.736, 0.617, and 0.827 for the 256-slice CT scanner, respectively. Thus, there was almost perfect agreement for the standard oblique algorithm for both CT scanners.

## Discussion

In the current work, the results obtained from this investigation indicated that the phenomenon of the five-line sign, which we identified previously using a 64-slice CT scanner [6], was present when using different types of CT scanners and under different reconstruction settings.

The phenomenon of the five-line sign or multi-line reflections, which appear within axial MIP reconstructions of interlobular fissures, may be caused by partial volume averaging in the lung [6, 8, 9] because the signs appear to be the beaded vessels exhibited by MIP-reconstructed pulmonary microvasculature, which was first proposed by Napel [8]. Evidence indicated that the beaded vessels and the multiple shadow lines of interlobular pleura belong to the same type of artificial shadows [6].

Of note, the 1.0-mm thin-slice is commonly used for three-dimensional reconstruction of lung microstructures and nodules [10–12]. We determined that the 1.0-mm thickness is the best for MIP-reconstruction of interlobular fissures, and that too thick or too thin slice thickness is not advisable [6]. In addition, the reconstruction space should be equal to the slice thickness. When the space is larger or smaller than the thickness, overlapping of multiple shadow lines or wider or narrower gaps between the lines can occur. Here, we used 1.0-mm thickness data derived from two CT scanners for oblique MIP reconstructions. In the Philips Brilliance™ Workspace Portal, the MIP reconstruction thickness must be increased to 6.0 mm before the five-line sign of interlobular fissures can be revealed.

In this study, high-resolution algorithms produced worse results than the standard-resolution algorithm with respect to presenting the five-line sign of the interlobular fissures. We reasoned that in comparison with a standard algorithm, the high-resolution method decreased the image smoothness and increased the sharpness, leading to a clearer microstructural edge in the lung parenchyma. However, this effect also increased image noise that may have overshadowed the density of the pulmonary microstructure.

The purpose of MIP reconstruction is to enhance the brightest pixels, thereby augmenting the contrast [8, 13]. When applying MIP reconstruction to study diffuse lung lesions, Remy-Jardin et al. [5] used high spatial-frequency reconstruction kernels to facilitate the reconstruction of transverse images in the lung window and noticed reduced intensity of the beaded vessels. This phenomenon was limited to the subpleural area and had poor imaging quality. This finding easily overcomes the challenge of distinguishing the string-of-beads artificial shadow of MIP-reconstructed microvasculature from the micronodules in diffuse lung lesions. Thus, the control of image noise is a key factor to determine the visibility of the five-line sign (Fig. 3b1-2). Remy-Jardin et al. [5] revealed some pulmonary micronodules that were less than 3 mm in diameter and had low density and a blurred boundary. MIP reconstruction with 8-mm thickness typically cannot reveal these microstructures, whereas 5-mm thickness is relatively ideal for displaying them. These findings indicate that increasing the MIP thickness may mask low-density structures in the lung, which is consistent with the observation in this study (Fig. 4).

When the standard axial algorithm was used for MIP reconstruction of the five-line sign, the low-clarity rates were 45% and 48% for the 16-slice and 256-slice CT scanners, respectively. In contrast, when the standard oblique algorithm was used, the low-clarity rates decreased to 0% and 2.7% for the 16-slice and 256-slice CT scanners, respectively, because the oblique lines rotated in areas where the oblique fissures were oriented at steep angles, such as the lower segments of the right oblique fissures and all three segments of the left oblique fissures. As a consequence, the oblique fissures and slices formed right angles that are exhibited as clear or barely clear in the most oblique multiplanar reformation images where they otherwise would have had an unclear axial MIP five-line sign, as shown in Fig. 2. In addition, for those areas where the five-line sign was clearly presented by the axial algorithm, the clarity level was not compromised by minor angle changes in the oblique plane. Under the generation of the five-line sign during the MIP reconstruction of interlobular fissures, the fissures and scanning plane must form a suitable angle, which can be easily accomplished by angle adjustment via multiplanar reformation on the sagittal plane of the workstation. The data in Table 4 show that the median of angles between the tangent lines and the fissures were − 27° to − 36° counter clockwise in most cases; the median of the right upper segments was the smallest, in most of which the five-line sign could be revealed in the axial MIP images directly (Table 3), and the median of the left lower segments was the biggest. The minor fissures were oriented at gentle angles with physical curvature (Fig. 2c), and the oblique lines had to be rotated clockwise or counter clockwise to form right angles between the oblique planes and fissures to show the five-line sign in some cases.

In most areas where the five-line sign could be discerned after adjustment of the oblique angle, the axial MIP images all contained band-like patterns with high density and clear boundaries. In comparison, in a few sites where the five-line sign was invisible after oblique angle adjustment, interlobular fissures in the axial MIP images were all manifested as a stripe-like pattern with low density and blurring boundaries.

This result was corroborated by the 2.7% of the samples derived from 256-slice CT and the standard oblique algorithm that failed to produce the five-line sign. These findings indicate that the proper density of interlobular fissures and suitable angles between the fissures and the section plane are necessary for the formation of the five-line sign. This requirement also explains why only the small blood vessels that are oblique to the MIP planes are manifested as strings of beads. Furthermore, the angle between the interlobular fissures and the scanning plane not only influenced the display rate of the five-line sign but also dictated the width of the lines [6]. Finally, in the lower right segment calculated with the high-resolution oblique algorithm, and the lower left segment calculated with the standard oblique algorithm, 256-slice CT displayed superior performance to 16-slice CT based on the rate of grading of the five-line sign as clarity level 3. This result may have been observed because imaging of the lower lobes of the lung was strongly affected by the heartbeat; therefore, 256-slice CT generated clearer images because of the faster scanning speed.

To outline the clinical usefulness of the five-line sign, we should consider that normal interlobular fissures feature regular morphology, smooth appearance, and uniform thickness. The MIP-rendered five-line sign has smooth lines and clear gaps and can, therefore, be used as the referencing object for other planar structures in the lung. Certain pulmonary pathological features, such as the pleural tail sign [14–16], represented a form of thickened septa [15]; the five-line sign revealed by MIP reconstruction suggested that it was a relatively smooth and regular plane, and it is difficult to determine using multiplanar or thin-section reconstruction. As we observed that most nodules with the pleural tail sign had an unclear or absent five-line sign, we speculated that the plane was rough and irregular; only a small number of nodules with the tail sign showed a clear five-line sign by MIP rebuilding, but these lines were thicker and denser than those of normal interlobular fissures. Simultaneously, it was noticed in our limited experiments that the tumour was at an advanced stage in most of the cases of lung cancer in which the five-line sign was unclear or absent, and relapse and metastasis usually occurred after resection [17]; whereas a small number of peripheral lung cancers with a clear five-line sign were at an earlier stage and no recurrence and metastases occurred following resection; these patients survived for more than 5 years disease-free [17]. In addition, a doubling time exceeding 800 days was observed in several tumours with a barely clear five-line sign and without experiencing being resected surgically. That implied character of indolent growth of those lung cancers with five-line sign. The cases of lung cancer mentioned involved almost completely solid nodules. Recent clinical studies reported the pleural tail sign or pleural indentation as having different clinical effects [16, 18, 19]; parts of these investigations focused upon pulmonary adenocarcinomas appearing as sub-solid nodules or ground-glass nodules [18, 19]. Other scholars have applied volume rendering reconstruction technique to predict whether the lung cancer invades the visceral or parietal pleura [20]. Anyhow, the authors of this article attempted to investigate the five-line sign of peripheral lung cancer through MIP reconstruction and to evaluate the effect of the five-line sign on staging and prognosis in peripheral lung cancer, especially when represented as a solid nodule; we consider that it would be worth carrying out further investigation on the basis of the large number of cases accumulated. This is the reason why we explored the five-line sign of the normal interlobular fissures using MIP reconstruction.

This study has limitations. The results were obtained from two different scanners only. Conventional scan parameters were used with half the subjects studied without tube current modulation. We did not obtain data on subjects scanned with both CT scanners (inter-individual and not intra-individual design).

In conclusion, the five-line sign of interlobular fissures can be revealed in MIP reconstructed images derived from different CT scanners. The standard oblique algorithm generated the best reconstruction outcome, whereas the clarity levels were not significantly different between the two CT scanners. The MIP-rendered five-line sign of normal interlobular fissures can be used as the referencing object for other planar structures in the lung.

## Abbreviations

CT: Computed tomography
MIP: Maximum intensity projection

## References

[1] Takahashi K, Thompson B, Stanford W (2006). Visualization of normal pulmonary fissures on sagittal multiplanar reconstruction MDCT. AJR Am J Roentgenol.

[2] Honda O, Johkoh T, Yamamoto S (2002). Comparison of quality of multiplanar reconstructions and direct coronal multidetector CT scans of the lung. AJR Am J Roentgenol.

[3] Remy-Jardin M, Campistron P, Amara A (2003). Usefulness of coronal reformations in the diagnostic evaluation of infiltrative lung disease. J Comput Assist Tomogr.

[4] Sakai M, Murayama S, Gibo M (2005). Can maximum intensity projection images with multidetector-row computed tomography help to differentiate between the micronodular distribution of focal and diffuse infiltrative lung diseases?. J Comput Assist Tomogr.

[5] Remy-Jardin M, Remy J, Artaud D, Deschildre F, Duhamel A (1996). Diffuse infiltrative lung disease: clinical value of sliding-thin-slab maximum intensity projection CT scans in the detection of mild micronodular patterns. Radiology.

[6] Yu A, Li Q, He J, Zhan Y (2014) Conditions and mechanism for the appearance of interlobar fissures as 5-line signs in axial maximum intensity projection images. J Comput Assist Tomogr 38:578–585. 10.1097/RCT.0000000000000085 24681848

[7] Landis JR, Koch GG (1977). The measurement of observer agreement for categorical data. Biometrics.

[8] Napel SA, Jeffrey RB, Fishman EK (1995). Principles and techniques of 3D spiral CT angiography. Spiral CT.

[9] Bhalla M, Naidich DP, McGuinness G et al (1996) Diffuse lung disease: assessment with helical CT--preliminary observations of the role of maximum and minimum intensity projection images. Radiology 200:341–47 10.1148/radiology.200.2.8685323 8685323

[10] Lee HY, Goo JM, Lee HJ (2009). Usefulness of concurrent reading using thin-section and thick-section CT images in subcentimetre solitary pulmonary nodules. Clin Radiol.

[11] National Lung Screening Trial Research Team (2011). The national lung screening trial: overview and study design. Radiology.

[12] van Klaveren RJ, Oudkerk M, Prokop M (2009). Management of lung nodules detected by volume CT scanning. N Engl J Med.

[13] Napel S, Rubin GD, Jeffrey RB (1993). STS-MIP: a new reconstruction technique for CT of the chest. J Comput Assist Tomogr.

[14] Hill CA (1982). “Tail” signs associated with pulmonary lesions: critical reappraisal. AJR Am J Roentgenol.

[15] Webb WR (1978). The pleural tail sign. Radiology.

[16] Hsu JS, Han IT, Tsai TH (2016). Pleural tags on CT scans to predict visceral pleural invasion of non-small cell lung cancer that does not abut the pleura. Radiology.

[17] Yu A, Li Q (2010) Role of the five line sign on peripheral lung cancer in diagnosis and prognosis. Paper presented at the 26th international congress of radiology (ICR 2010), Shanghai, China, 9−10 April 2010

[18] Zhang Y, Shen Y, Qiang JW (2016). HRCT features distinguishing pre-invasive from invasive pulmonary adenocarcinomas appearing as ground-glass nodules. Eur Radiol.

[19] Zhao LL, Xie HK, Zhang LP (2016). Visceral pleural invasion in lung adenocarcinoma ≤3 cm with ground-glass opacity: a clinical, pathological and radiological study. J Thorac Dis.

[20] Ebara K, Takashima S, Jiang B (2015). Pleural invasion by peripheral lung cancer: prediction with three-dimensional CT. Acad Radiol.

